# Challenges in tendon–bone healing: emphasizing inflammatory modulation mechanisms and treatment

**DOI:** 10.3389/fendo.2024.1485876

**Published:** 2024-11-06

**Authors:** Fan Jiang, Haibo Zhao, Po Zhang, Yanchi Bi, Haoyun Zhang, Shenjie Sun, Yizhi Yao, Xuesai Zhu, Fenghua Yang, Yang Liu, Sicong Xu, Tengbo Yu, Xiao Xiao

**Affiliations:** ^1^ Department of Orthopedic Surgery, Qingdao Municipal Hospital, Qingdao University, Qingdao, China; ^2^ Department of Orthopedic Surgery, Qingdao Municipal Hospital, University of Health and Rehabilitation Sciences, Qingdao, China; ^3^ Central Laboratories, Qingdao Municipal Hospital, University of Health and Rehabilitation Sciences, Qingdao, China

**Keywords:** inflammation, macrophage, fibroblasts, tendon-bone healing, biomaterials

## Abstract

Tendons are fibrous connective tissues that transmit force from muscles to bones. Despite their ability to withstand various loads, tendons are susceptible to significant damage. The healing process of tendons and ligaments connected to bone surfaces after injury presents a clinical challenge due to the intricate structure, composition, cellular populations, and mechanics of the interface. Inflammation plays a pivotal role in tendon healing, creating an inflammatory microenvironment through cytokines and immune cells that aid in debris clearance, tendon cell proliferation, and collagen fiber formation. However, uncontrolled inflammation can lead to tissue damage, and adhesions, and impede proper tendon healing, culminating in scar tissue formation. Therefore, precise regulation of inflammation is crucial. This review offers insights into the impact of inflammation on tendon–bone healing and its underlying mechanisms. Understanding the inflammatory microenvironment, cellular interactions, and extracellular matrix dynamics is essential for promoting optimal healing of tendon–bone injuries. The roles of fibroblasts, inflammatory cytokines, chemokines, and growth factors in promoting healing, inhibiting scar formation, and facilitating tissue regeneration are discussed, highlighting the necessity of balancing the suppression of detrimental inflammatory responses with the promotion of beneficial aspects to enhance tendon healing outcomes. Additionally, the review explores the significant implications and translational potential of targeted inflammatory modulation therapies in refining strategies for tendon–bone healing treatments.

## 1 Introduction

Tendons, vital structures composed of fibrous connective tissue, serve as the bridges that transmit the powerful forces generated by muscles to bones (1). While tendons exhibit remarkable resilience to various stresses, they are also susceptible to significant damage. Compounded by their limited regenerative capacity due to a lack of vascular supply, repairing tendons poses a formidable challenge in the realm of medical science (2). Annually, the United States witnesses over 300,000 cases necessitating surgical interventions for tendon or ligament injuries—a number that continues to ascend in line with population growth and the burgeoning enthusiasm for sports activities (3). These injuries often manifest as either degenerative chronic conditions or acute traumas resulting from direct ruptures, both of which profoundly alter the structural integrity and functionality of tendons.

Among the most prevalent conditions requiring tendon grafts are rotator cuff tears (RCTs) and anterior cruciate ligament (ACL) injuries. Given the similarities in their structures, the healing of ligaments is frequently compared to and associated with tendon healing. This parallel often leads to significant pain, functional impairment, and the potential for disability. The rotator cuff, an assembly of tendons from muscles like the supraspinatus, infraspinatus, subscapularis, and teres minor, stands as a classic example of a tendon structure prone to injury. Statistical data reveal that more than 270,000 surgeries are performed annually in the US to rectify torn rotator cuff tendons (4). Unfortunately, despite advancements in surgical techniques, retear rates remain alarmingly high, particularly in cases of larger tears, where retear rates can soar to 94% (5). Even with the application of improved treatment modalities encompassing nonoperative, operative, and rehabilitative approaches, outcomes remain inconsistent, with healed tendon tissue seldom regaining its pre-injury functionality (6, 7) and sometimes experiencing up to a 30% decrease in ultimate tensile strength post-healing (8).

The process of tendon-to-bone healing, a critical facet of tendon recovery, unfolds in four distinct phases: inflammation, proliferation, remodeling, and maturation. However, given the inherently low vascularity and metabolic activity within tendons, traditional treatments often fall short of fully restoring tendon structure and function, potentially leading to complications such as tendon adhesion and bone resorption. Consequently, achieving efficacious tendon-to-bone healing emerges as a pivotal yet formidable aspect of tendon recovery (9). The native bone-to-tendon interface (BTI) intricate transition from tendon to non-mineralized fibrocartilage, mineralized fibrocartilage, and ultimately bone plays a pivotal role in stress transfer and the prevention of stress concentrations (10, 11). The quality of tendon–bone healing plays a crucial role in determining the likelihood of retear following rotator cuff repair. During the healing process, fibrous scar tissue tends to replace the original uncalcified and calcified fibrocartilage at the tendon-to-bone interface (12–15). While surgical procedures can reestablish continuity between tendon and bone, they frequently struggle to regenerate the native multi-tissue transition, impacting stress distribution and heightening vulnerability to retear (16).

**Figure 1:**
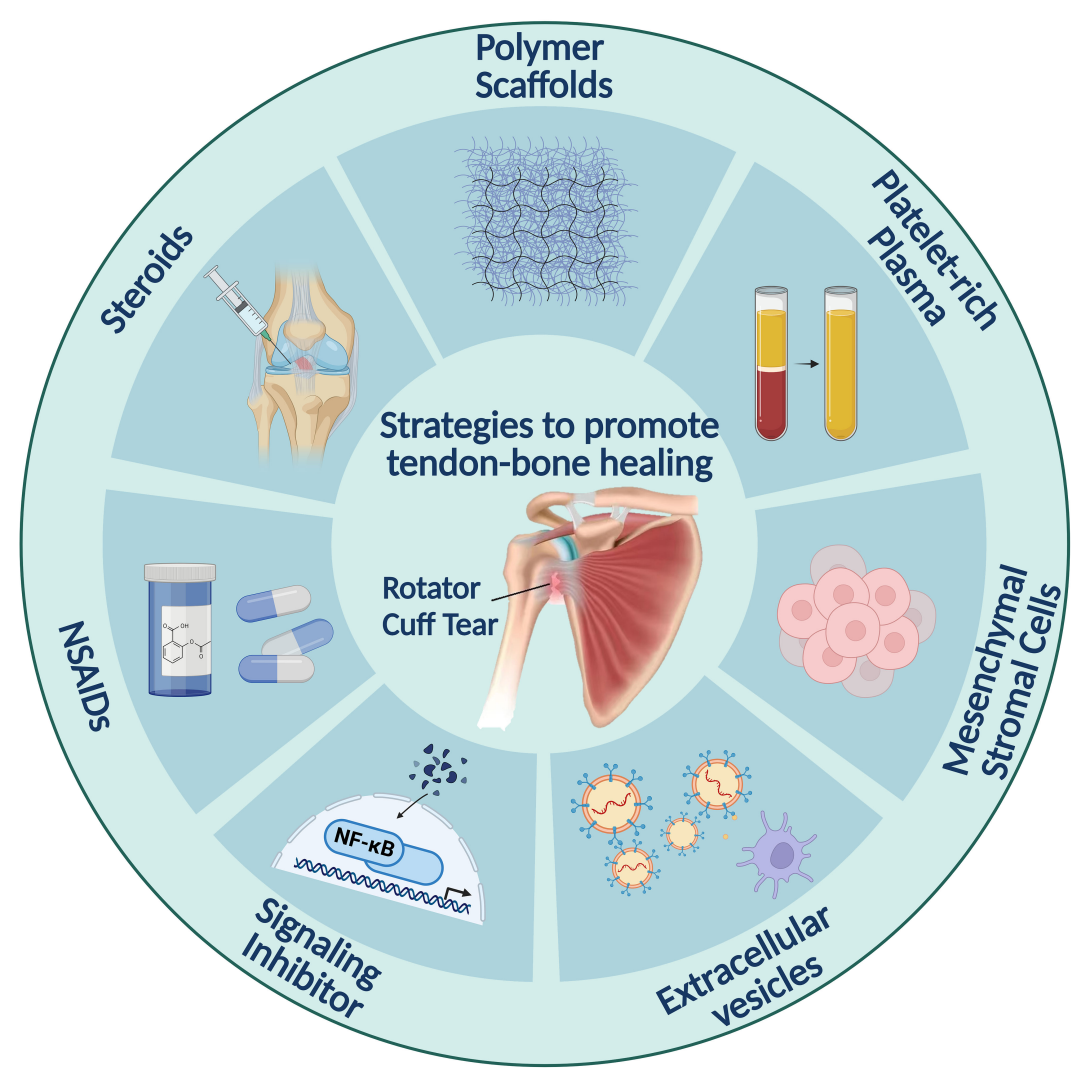
Strategies for promoting tendon–bone healing through inflammation regulation. Polymer scaffolds provide the structural framework for tissue regeneration. The platelet-rich plasma then provides growth factors to stimulate cell growth and differentiation. Steroids and NSAIDs are used to control inflammation. Mesenchymal stromal cells and signaling inhibitors target specific immune pathways. Extracellular vesicles promote intercellular communication and regulate cellular processes involved in healing.

Inflammation is a necessary stage in tendon healing. The inflammatory microenvironment attracts immune cells such as macrophages, which help remove debris and release growth factors that initiate the healing process. Although inflammation is necessary to initiate the healing process, excessive inflammation can lead to deleterious effects such as scar tissue formation and impaired tissue regeneration (17). Therefore, controlling inflammation during the healing process is essential to promote optimal tendon repair. This review primarily focuses on the role of cellular or molecular responses during the inflammation phase in tendon–bone healing, exploring potential signaling pathways, immune cell activation, and therapeutic strategies to optimize outcomes. By elucidating the inflammatory microenvironment and the intricate interactions among different cell types and the extracellular matrix (ECM), we aim to develop more effective approaches to promote tendon–bone healing. This review also explores the beneficial mechanisms by which biomaterials promote tendon–bone healing and repair through their immunomodulatory effects, and develops innovative therapeutic strategies by investigating the use of biomaterials in tendon injuries (**Figure 1**), aiming to provide more effective options for patients with tendon injuries.

## 2 Overview of tendon–bone healing

### 2.1 Physical structure of a tendon–bone insertion

The musculoskeletal system relies on the coordination of various connective tissues, including the specialized tendon–bone insertion where tendons or ligaments attach to bone (18). The rotator cuff commonly experiences tears at its tendon–bone insertion site (19). The enthesis, where soft tissue anchors to the bone, acts as a transitional zone between tendon and bone, exhibiting tapered mechanical properties. This region houses specialized cell types like osteoblasts, osteocytes, osteoclasts, fibrochondrocytes, and tenocytes. The enthesis is segmented into four zones: tendon, non-mineralized fibrocartilage, mineralized fibrocartilage, and bone zones (20). Each zone possesses distinct compositions crucial for stress distribution and energy transfer, aiding in minimizing stress forces transmitted between soft and hard tissues (21). The fibrocartilage zone absorbs stress concentrations, the demineralized region withstands pressure, and the mineralized region protects bone from shear stress (22). The transitional zone fosters cellular interactions for maintaining function, with variations in elastic moduli posing challenges for biomaterial replication due to the intricate nature of the interface.

### 2.2 Healing processes of tendon–bone insertion

Tendon healing post-injury progresses through three overlapping stages: inflammation, proliferation, and remodeling (23). Initially, blood accumulates and clots at the injury site, triggering platelet degranulation and release of cytokines and growth factors. The subsequent inflammatory stage, lasting 3 to 7 days, attracts inflammatory cells like neutrophils, monocytes, and macrophages, crucial for clearing debris and promoting healing at the tendon–bone interface. Various growth factors aid in tissue repair, with vascular growth factor induction ensuring adequate blood supply to the newly formed tissue (24, 25). The proliferation stage involves fibroblast infiltration, collagen deposition, and stem cell activities, culminating in vascular remodeling (26). During remodeling, a new ECM is synthesized, bone ingrowth occurs, and collagen continuity is established between the tendon graft and bone (27). Maturation sees reduced cells and blood vessels, collagen fiber orientation, and gradual restoration of biomechanical strength, albeit with inferior quality compared to uninjured tendons (28).

### 2.3 Understanding the mechanisms of tendon–bone healing: intrinsic and extrinsic factors

Tendon healing involves intrinsic and extrinsic mechanisms, with intrinsic and extrinsic cell populations playing vital roles. Studies on tendon healing during embryonic development highlight the importance of understanding intrinsic and extrinsic cell activities, crucial for scar formation and adhesion prevention (29). Extrinsic healing involves infiltrating sheath and synovium cells, leading to collagen III-rich scar formation, while intrinsic healing recruits tenocytes to promote collagen I fiber secretion, enhancing mechanical properties (23). The healing process typically lasts up to 12 months, with collagen III fibers gradually replaced by collagen I fibers, posing a risk of retearing during the initial postoperative year (30–32).

## 3 Challenges in tendon–bone healing: inflammation, scar formation, and vascularization

The native insertion site at the tendon–bone interface features a complex layered structure crucial for its unique functionality. However, injured tendon tissue encounters substantial hurdles in regaining its anatomical integrity. Early robust inflammatory responses often precipitate adhesion and scar formation, weakening the tendon and heightening the risk of retearing (33). Notably, cytokines play a pivotal role in this intricate healing process. Post-surgical reconstruction, an array of pro-inflammatory factors accumulates in the surrounding tendon tissue, leading to tissue necrosis, collagen disarray, myotendinous degeneration, and impeded tendon neovascularization. Most tendon-to-bone healing processes grapple with insufficient vascularization at the tendon–bone interface, curtailing oxygen supply to cells, affecting the production of multiple growth factors, and hampering cell proliferation (34). Furthermore, tendons are vulnerable to the adverse effects of excessive loading, resulting in limited self-repair and regenerative capabilities (35), ultimately culminating in sluggish or unsuccessful tendon–bone healing (36). Presently, no strategies exist to bolster the anatomical regeneration of the tendon enthesis. Therefore, a primary focus in the realm of regenerative medicine is to enhance the functionality and biological recuperation of tendon–bone injuries. The principal objectives in treating tendon injuries encompass anti-inflammatory measures, scar formation inhibition, and the promotion of vascularization (37, 38).

### 3.1 Role of inflammation in tendon–bone healing and fibrosis: insights into the microenvironment

Tendon–bone healing is a multifaceted process influenced by tear severity and microenvironment stability, spanning months to years. The inflammatory cascade ignited post-hemostasis and clotting assumes a pivotal role in orchestrating healing as a defensive response to cellular insult (39). Despite the manifold functions of inflammation in tissue regeneration, it primarily operates as a negative regulator of tendon–bone healing. Dysregulated inflammation has the potential to disrupt the tendon matrix microenvironment, impeding homeostasis (40).

Clinical cases (41) and animal model studies (42) have evidenced that inflammatory reactions correlate with a notably higher retear rate and compromised histological scores at the tendon–bone interface. Excessive scar tissue formation and compromised biomechanical properties ensue following an inflammatory response to a tendon–bone junction injury. Advanced methodologies have further substantiated inflammation in diverse tendon injuries, underscoring its involvement in their onset and progression (43, 44). Excessive inflammation can also dictate the type of tissue healing, fostering fibrosis over tissue regeneration.

Apart from alterations in the tendon ECM and cellular milieu, patients with systemic comorbidities that intensify the inflammatory response during healing face an elevated risk of pathological fibrosis post-tendon injury (45). Substantial evidence supports the premise that inflammation significantly impedes tendon healing, particularly in individuals with obesity and type 2 diabetes mellitus (T2DM) (46). The modified composition of the tendon ECM and the presence of heightened pro-inflammatory cytokines in these patients prolong the inflammatory response, ultimately culminating in fibrosis during the healing journey. Macrophages, integral components of the immune system, play a pivotal role in tendon healing. However, in obese and T2DM patients, there exists a bias in macrophage polarization towards the pro-inflammatory M1 phenotype (47, 48). This skewed polarization perpetuates the inflammatory response, fostering fibrotic changes within the tendon. Investigations on rodent models of obesity and T2DM have unveiled augmented fibrosis and significantly impaired mechanical properties in flexor tendons, highlighting the impact of these comorbidities on healing outcomes (49–51). Notably, experiments on obese and T2DM mice have revealed sustained expression of M2 macrophage markers alongside elevated expression of M1 macrophage markers throughout the healing process (49). This sustained inflammation disrupts the normal healing cascade, protracting the pro-inflammatory milieu. Consequently, impaired healing responses contribute to the development of fibrotic tendon disorders and complications in patients with obesity and/or T2DM. While the precise reasons for subpar tendon healing in these individuals may be multifaceted, evidence robustly underscores the role of inflammation in propelling tendon fibrosis (52). Understanding the interplay among systemic comorbidities, inflammation, and fibrosis in tendon healing is imperative for devising targeted therapeutic interventions that can ameliorate outcomes for patients with obesity and T2DM.

In essence, patients with obesity and T2DM face heightened challenges in tendon healing due to alterations in the tendon ECM, elevated levels of pro-inflammatory cytokines, biased macrophage polarization, increased fibrosis, and impaired mechanical properties (53). The sustained expression of macrophage markers throughout the healing process further underscores the critical role of inflammation in driving tendon fibrosis. Upholding a balance between inflammatory activation and inhibition is essential for effective healing, considering the interplay among inflammatory and non-inflammatory cells, as well as the ECM (54). A comprehensive understanding of the inflammatory microenvironment is pivotal for fostering optimal healing outcomes in tendon–bone injuries, offering insights into the mechanisms underpinning fibrosis and impaired healing. By addressing the impact of inflammation and factoring in systemic comorbidities, strategies can be formulated to modulate the inflammatory response and augment tendon–bone healing.

### 3.2 Role of immune cells in the inflammatory microenvironment

In the inflammatory microenvironment, immune cells play a pivotal role in orchestrating the cascade response crucial for tendinopathy initiation and progression, alongside other related conditions (40, 55). Upon the surgical attachment of a tendon to a bone, the initial cellular response predominantly involves the infiltration of inflammatory cells (44). Hays et al. employed a rat model of ACL reconstruction to study the healing process of a tendon graft within a bone tunnel. They observed an initial infiltration of inflammatory cells, followed by the development of a loosely organized and disordered fibrous tissue interface at the tendon–bone junction (56). Neutrophils, macrophages, and lymphocytes, in a specific temporal and spatial sequence, respond to injury signals and are recruited from circulation to the tendon–bone interface (45). Moreover, resident immune cells such as mast cells, dendritic cells, and T lymphocytes actively contribute to the inflammatory microenvironment and immune regulation post-tendon injuries (57). The roles of immune cells in tendon injury are shown in **Table 1**.

**Table 1:** Key immune cells involved in tendon inflammation.

Immune Cell	Role	Mechanisms	Research Findings	Clinical Significance	References
Neutrophils	Initial responders, remove debris, triggering inflammation	Phagocytosis, ROS production, and NET formation	Elevated in tendinopathy models, involved in both innate and adaptive immune responses	Excessive accumulation can lead to tissue damage	(58)
Macrophages (M1/M2)	Promote inflammation (M1) and facilitate tissue repair (M2)	Release cytokines and regulate ECM	M1–M2 balance crucial for healing, M2 associated with fibrosis	Modulating macrophage polarization is a therapeutic target	(59)
T lymphocytes	Regulate inflammation and tissue remodeling	Secrete cytokines (Th1/Th2/Tregs)	Involved in scar formation and tendon regeneration	Targeting T-cell subsets may be beneficial	(60)
Mast cells	Mediate inflammation, regulate collagen turnover, and interact with nerves	Release histamine, proteases, and growth factors	Increased tendinopathy and influences vascular growth and pain	Potential therapeutic target	(61)
Natural killer (NK) cells	Role in tendon injury unclear	May influence inflammation and tissue repair	Found in chronic tendinopathy	Further research needed to understand their role	(62)

ECM, extracellular matrix; NETs, neutrophil extracellular traps; ROS, reactive oxygen species; Th1, T helper 1 cell; Th2, T helper 2 cell; Tregs, regulatory T cells.

#### 3.2.1 Neutrophils

Neutrophils, as the first responders to tissue injury, play a critical role in the inflammatory response. Their functions include removing bacteria and necrotic tissue through phagocytosis and the release of protease-containing granules. They also generate a hydrogen peroxide gradient via enzyme complexes like NADPH oxidase and ROS, which are essential for initiating the inflammatory cascade (63). However, the formation of neutrophil extracellular traps (NETs) by activated neutrophils requires tight regulation to prevent excessive tissue damage due to their cytotoxic potential (64). Additionally, neutrophils can inhibit healing by releasing soluble mediators, excessive ROS, and pro-inflammatory microRNAs contained in granules, leading to tissue damage (65). Efficient healing relies on the clearance of neutrophils from the inflammatory microenvironment. Neutrophils undergo apoptosis or necrosis and are engulfed by macrophages through phagocytosis or efferocytosis to maintain cellular homeostasis. Some neutrophils may leave the injury site through reverse migration or return to the circulatory system (44). However, failure to clear neutrophils often leads to secondary necrosis, resulting in tissue damage and sustained inflammation through the release of cytotoxic, pro-inflammatory, and immunogenic molecules by the lysing cells (66).

While their involvement in general tissue healing is well-established, their specific role in tendon injury has been a subject of growing interest. Recent studies have highlighted the presence of neutrophils in various tendinopathy models, suggesting their potential contribution to tendon pathology. Published studies have revealed the role of neutrophils in tendon injury. For example, the study by Marsolais et al. demonstrated a sequential accumulation of neutrophils and macrophages in Achilles tendon injury models. Neutrophils were initially elevated, followed by a surge in macrophages, suggesting a coordinated inflammatory response (67). A study by Crowe et al. found that alarm molecules such as S100A8 and S100A9 are upregulated in tendinopathic tissues and that these molecules recruit immune cells such as neutrophils, further exacerbating the inflammatory response (68). Noah et al. observed a robust adaptive immune response in tendons following injury, preceded by an initial innate immune response involving neutrophils and macrophages. This suggests a complex interplay between innate and adaptive immune cells in tendon healing (58). Ribitsch et al. showed differences in neutrophil responses in tendon injury models in fetal and adult animals. Tendon injury in adult animals was accompanied by a stronger inflammatory response and more neutrophil infiltration, whereas the inflammatory response was weaker during the regeneration of fetal tendons (69). These studies provide valuable insights into the nuanced role of neutrophils in tendon injury and provide a basis for exploring whether excessive neutrophil accumulation uniquely contributes to tendon pathology.

In summary, neutrophils play a multifaceted role in tendon injuries. Although neutrophils are essential for removing debris and triggering inflammation, excessive accumulation of neutrophils can lead to tissue damage and delayed healing. Understanding the mechanisms by which neutrophils are involved in these processes is essential for the development of effective therapeutic interventions. Future research should focus on exploring the specific role of neutrophils in scar formation during tendon healing and developing strategies to regulate their activation, migration, and clearance.

#### 3.2.2 Macrophages

Macrophages play a critical role in tendon–bone healing. Macrophages, the predominant subset of immunocytes infiltrating pathologic tendons, play a crucial role in regulating local inflammation. Macrophages, among the various immunocytes, have been observed to infiltrate the damaged tendon, orchestrating local inflammation and regulating the healing process (59). Wong et al.’s model of tendon adhesion showed that there is a large infiltration of inflammatory cells into subcutaneous tissue and tendon wounds within 24 h of tendon injury (70). Injured tendon tissue releases chemokines, with CCL2 recruiting macrophage-dominated immune cells (71). Marsolais et al. demonstrated that in an Achilles tendon injury model, the influx of macrophages leads to further release of chemokines and cytokines, followed by a macrophage surge, suggesting that a synergistic inflammatory response exists and plays a critical role (67).

**Figure 2:**
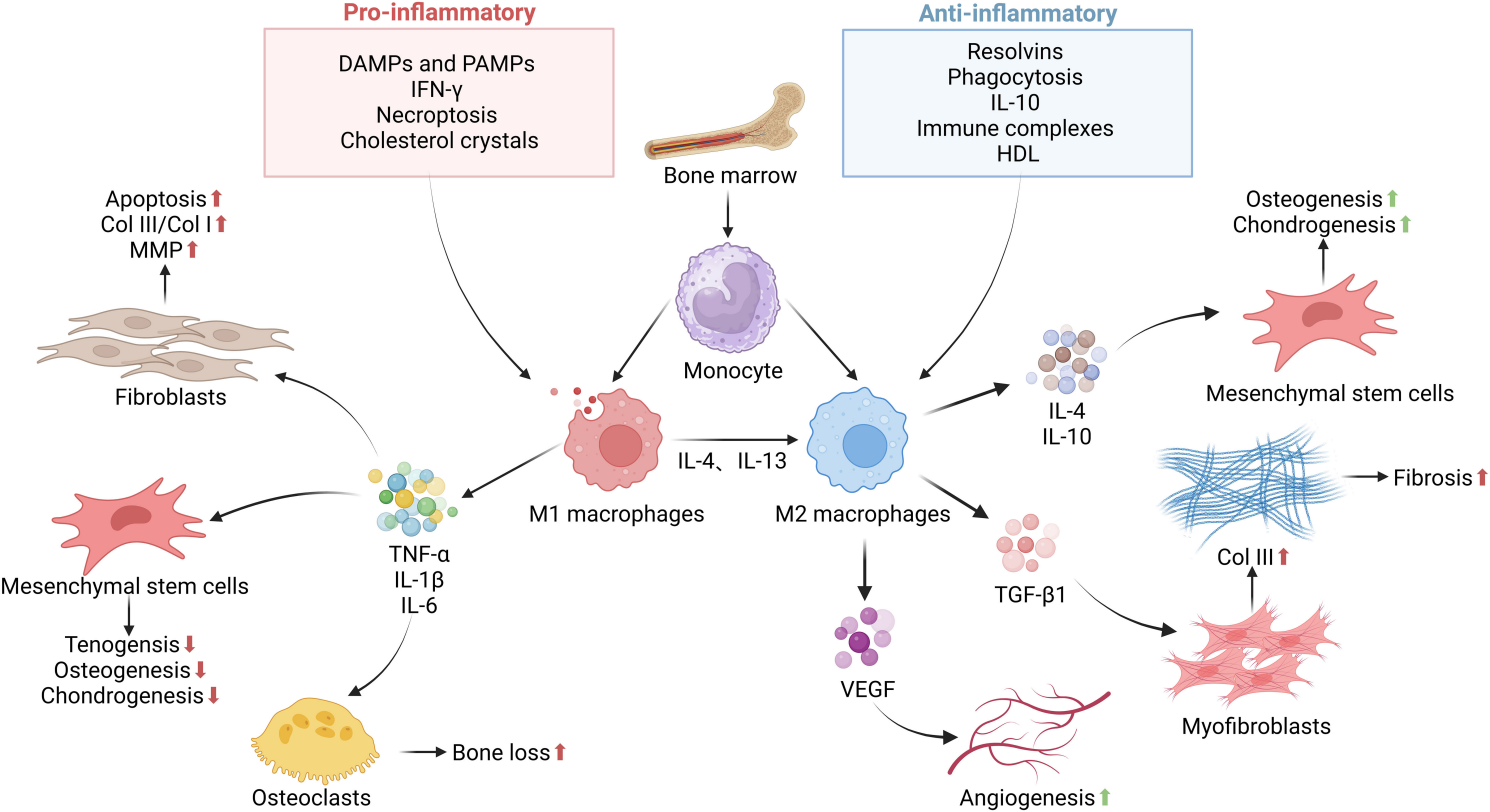
The functions and regulation of macrophages in tendon–bone healing. The inflammatory phase as a key step in determining normal or impaired tendon–bone healing. This stage is essential for the clearance of bacteria, tissue debris, apoptotic cells, and clots from the wound site. Macrophages are recruited from the bone marrow to the tendon–bone interface, where recruited and resident macrophages undergo significant phenotypic and functional changes mediated by DAMPs, PAMPs, growth factors, cytokines, and other mediators present in the local tissue microenvironment. Upon specific stimuli, M0 macrophages can polarize into M1 and M2 macrophages, releasing pro-inflammatory, anti-inflammatory, and growth factors that influence the proliferation, differentiation, and activation of fibroblasts, mesenchymal stem cells, and other cells involved in tissue repair. In the later stages of the repair process, they exhibit a reparative regulatory phenotype to suppress the inflammatory response to tissue damage and restore normal tissue structure. Failure to effectively control this process can lead to persistent inflammation and/or maladaptive repair, resulting in destructive tissue fibrosis.

Macrophages exhibit significant functional diversity, classified as proinflammatory M1 and anti-inflammatory M2 phenotypes (72–74). They exhibit a phenotypic shift from M1 pro-inflammatory to M2 anti-inflammatory macrophages during tissue repair (75), releasing different cytokines that contribute to tissue regeneration and wound repair (59, 76). During the early stages of tendon healing, infiltrating macrophages at the site of injury are predominantly of the M1 phenotype (77), and their concentration increases significantly during the first 2 weeks of tendon healing and is localized to newly formed tendon tissues and areas of tissue remodeling (71), where the M1 macrophages migrate to the site of injury and release pro-inflammatory cytokines, such as interleukin-1 (IL-1), tumor necrosis factor-α (TNF-α), and interleukin-6 (IL-6), which may hinder tissue repair (71). Conversely, M2 macrophages are primarily involved in the later stages of healing, demonstrating the ability to eliminate intracellular pathogens and promote tendon regeneration (74, 78). The balance between M1 and M2 macrophages is crucial for successful wound healing (**Figure 2**). In a previous study, Kawamura et al. examined the interface between the free flexor digitorum longus tendon and bone tunnel and observed that M1 macrophages were present early after surgery and persisted for 4 weeks. In contrast, M2 macrophages were not detected until 11 days after surgery and exhibited high expression levels up to 4 weeks (44). Thus, regulating the transition of macrophages from the M1 to M2 state plays a crucial role in improving tendon–bone healing. Chronic inflammatory models have provided evidence linking the failure of macrophages to transition from M1 to M2 phenotypes with delayed healing and persistent shoulder pain following treatment. For instance, Dakin et al. discovered that the expression of CD206, a cell surface marker associated with M2 macrophages, was higher in pain-free patients after surgical subacromial decompression compared to those experiencing ongoing pain (79). Similarly, Chamberlain et al. demonstrated in a mouse model of Achilles tendon rupture that a decreased M1/M2 ratio promoted the biological response necessary for tendon healing (80). Consequently, modulating the balance between M1 and M2 macrophages presents an appealing target for treating tendinopathy.

Impaired switching from M1 to M2 macrophages can result in poor wound closure and chronic inflammation (73). Dagher et al. performed a study and found a reduction in M1 macrophage aggregation at the tendon–bone interface after 2 and 4 weeks of postoperative fixation. This reduction was associated with improved histological and biomechanical properties at the adhesion (81). In another study by Gulotta et al., after administration of TNF-α antibody in a rat rotator cuff injury model, there was a significant reduction in M1-type macrophages and improved tendon–bone healing (82). The above studies suggest that enhanced macrophage polarization from M1 to M2 accelerates tendon–bone healing and that control of macrophage polarization is essential to prevent tissue damage caused by the prolonged presence of M1 macrophages and to avoid dysregulated or delayed M2 polarization, which leads to chronic inflammation and impaired wound healing.

In contrast to M1 macrophages, M2 macrophages are thought to be involved in the promotion of fibroblast proliferation and new tissue deposition, which are essential for the regeneration and remodeling of tendon tissue (83). The concentration of M2 macrophages increases significantly during the later stages of tendon healing, particularly in the area where the tendon ECM is located (84). It has been shown that 28 days after tendon injury, M2 macrophages increase substantially and become the predominant macrophage phenotype at the site of injury (71). Appropriate mechanical stimulation has been found to promote macrophage polarization towards the M2 phenotype and secretion of high levels of TGF-β1 to promote chondrogenic differentiation of mesenchymal stem cells (MSCs), thereby enhancing tendon–bone healing (85). Although M2 macrophages are critical for anti-inflammatory responses and tissue remodeling in the early stages of tendon repair, their long-term dominance in the later stages may promote exogenous healing, potentially leading to the formation of fibrotic scar tissue (49). Recent studies have shown that M2-type macrophages can also be pro-fibrotic. For example, an increase in M2-type macrophages has been associated with the development of pulmonary fibrosis, and reducing the accumulation of M2-type macrophages has been shown to attenuate fibrosis in a mouse model of idiopathic pulmonary fibrosis (86). These observations have been further validated by studies in patients with hypertrophic scars, whose skin showed significantly more M2 phenotypic macrophages even before the onset of trauma compared to patients with normal scars (87). As in other tissues, M2-type macrophages can be pro-fibrotic in tendons. In tendon injury, TGF-β1 derived from M2 macrophages may also lead to tendon adhesion, recruit MSCs, and promote myofibroblast formation in tendon adhesion (88). During diabetic tendon healing, overproduction of TGF-β1 by M2 macrophages can lead to pathological fibrosis (89). These findings suggest that M2 macrophages play a facilitating role in fibrosis. In conclusion, the role of M2 macrophages in tendon healing is complex, affecting both intrinsic and extrinsic healing processes. Balancing macrophage activity is essential to support intrinsic healing while attenuating excessive exogenous tissue formation. Precise temporal and spatial regulation of macrophage phenotypes is essential for optimal tendon recovery and the development of effective tendon injury treatment strategies.

In order to establish a causal relationship between macrophages and tendon–bone healing, Hays et al. conducted a study using liposomal clodronate to eliminate macrophages in rats. Their findings demonstrated improved tendon–bone healing at the graft and bone tunnel site (56, 90). Subsequently, several studies focused on developing various modalities targeting macrophages to enhance tendon–bone healing. One such modality was the use of an electrospun nano-scaffold developed by Song et al., which exhibited the ability to inhibit macrophage accumulation. In a rat model of rotator cuff injury, this scaffold was shown to promote tendon–bone healing (90). Another approach was taken by Lu et al., who utilized fresh bone marrow to facilitate the M2 polarization of macrophages. This intervention resulted in the formation of better-organized connective tissue between the tendon and bone (91). These studies highlight the significance of macrophages in tendon–bone healing and demonstrate the potential of macrophage-targeting strategies to promote this process.

Successful tendon–bone healing after tendon injury repair requires both mechanical and biological enhancement. Macrophages play a crucial role in the inflammatory response of the tendon-bone interface induced by surgery, which can inhibit tendon-bone healing. (92). M1 macrophages are initially present and exhibit proinflammatory functions, while M2 macrophages gradually replace them with anti-inflammatory functions. Accelerating the transition from M1 to M2 polarization of macrophages can expedite tissue repair. Failure to convert macrophages to the M2 phenotype in a timely manner can lead to prolonged local inflammation and delayed healing (93). Therefore, modulating the polarization state of macrophages may be a valuable strategy to promote early healing in tendon injury repair. Macrophages play a vital role in the inflammatory response and subsequent wound-healing processes. Their timely polarization from proinflammatory M1 to anti-inflammatory M2 phenotypes is critical for effective tissue repair and regeneration. Failure to regulate macrophage polarization can lead to chronic inflammation, impaired healing, and excessive scar formation. Understanding the role of macrophages in tendon–bone healing after tendon injury provides insights into potential therapeutic strategies for promoting efficient and accelerated healing in these conditions.

#### 3.2.3 T lymphocytes

The accumulation of T cells in tendon injuries and diseases suggests their potential role in the healing process. In spondyloarthritis, a specific type of tendon-specific T cell characterized by RORγt expression has been identified, lacking CD4 and CD8 markers (94). These T cells respond to IL-23 and secrete IL-22, stimulating osteoblast-mediated bone remodeling and promoting bone formation. Additionally, muscle-specific regulatory T cells (Tregs) influence tissue repair by reducing inflammation and secreting factors that directly impact satellite cell growth (95).

Following tendon injury, CD4+ T cells migrate to the tissue, releasing pro-inflammatory cytokines including IL-2 to enhance the innate immune response for tendon repair. The phenotypic heterogeneity of immune cell populations at the tendon–bone interface underscores the importance of understanding their specific functions (96). Th2 cells are associated with promoting collagen synthesis and fibronectin deposition, while Th1 cells alleviate fibrosis by inhibiting fibroblast proliferation and collagen gene expression (97).

Interestingly, recent studies using *in vitro* culture systems have revealed a positive inflammatory feedback loop between tendon cells and T cells, although the specific subtypes of T cells remain undetermined (60). While these studies suggest a potential pathological role of T cells in tendon healing, recent findings indicate that neonatal tendon regeneration relies on Tregs, as depletion of Tregs leads to impaired structural and functional healing. Unlike adult Tregs, neonatal Tregs promote regeneration by shifting macrophages from pro-inflammatory to anti-inflammatory phenotypes (98). Transfer of neonatal Tregs to adult hosts improves macrophage polarization and consequently facilitates functional recovery. Moreover, various injury models suggest a protective role of IL-33 in promoting Treg expansion across multiple tissues, although it may also exhibit pathological effects (99).

In conclusion, these research findings highlight the impact of T cells on tendon–bone healing and underscore the importance of comprehensively understanding their contributions to strengthening therapeutic strategies.

#### 3.2.4 Mast cells

Mast cells play a crucial role in tendon healing and are usually located locally in tendon tissue or adjacent connective tissue, such as tendon, muscle–tendon junction, or bone–tendon junction. They can migrate to the site of injury in response to inflammation and nerve growth (100). It is uncertain whether the increase in mast cells at injured tendon sites is entirely due to migration or whether mast cells also proliferate or originate from circulating progenitor cells (101). The complex and fascinating role of mast cells in tendinopathy is a subject of ongoing research.

Activated mast cells release a variety of pre-formed and synthesized bioactive substances, including vascular endothelial growth factor (VEGF) and nerve growth factor (NGF), which contribute to new blood vessel formation and nerve growth during tendon healing (102). Mast cells also regulate collagen renewal, a key process in tendon tissue repair. By producing growth factors such as TGFβ and FGF2, mast cells stimulate collagen synthesis in fibroblasts. Mast cell-derived proteases (e.g., trypsin-like and rennet) can further enhance collagen synthesis in fibroblasts and may have similar effects on tendon cells (103). In addition, mast cells activate matrix metalloproteinases that promote collagen degradation. The proximity of mast cells to peripheral nerve endings suggests that they can be activated by neurotransmitters released by nerves after tendon injury (104).

Mast cells express receptors for various neurotransmitters such as substance P, glutamate, CGRP, and neurokinin A, suggesting their responsiveness to these signaling molecules (105). Activation of mast cells by neurotransmitters leads to the release of cytokines and other pro-inflammatory mediators that may contribute to the pathology of tendon injury (106). In addition, mast cells can activate peripheral nerve cells by secreting neurotransmitters such as histamine, serotonin, and dopamine. Upregulation of histamine receptor 1 in injured nerve fibers may be associated with neuropathic pain (53).

Clinical studies have shown an increased number of mast cells in tendinopathic tissues compared to healthy tissues, especially within injured tendons (61). Mast cells are closely associated with neovascularization, suggesting their influence on vascular growth in inflamed tendons (107). The density of mast cells correlated with the area of blood vessels and higher mast cell numbers were observed in patients with longer duration of symptoms (107). Similar findings were found in animal models of tendon injury and tendinopathy (106, 108, 109).

In conclusion, mast cells play a complex role in the inflammatory process of tendon healing. They migrate to the site of injury, release bioactive compounds, regulate collagen renewal, respond to neurotransmitters, and interact with nerve cells. Understanding the involvement of mast cells in tendon pathology may pave the way for potential therapeutic interventions to treat tendon injuries. Further studies are needed to delve into the specific role of mast cells in the healing process after tendon injury.

#### 3.2.5 Natural killer cells

Natural killer (NK) cells also play an important role in tissue injury repair (110). However, other studies have shown that NK cells may cause delayed wound healing due to the production of pro-inflammatory cytokines (62). Studies have shown that more NK cells are found in chronic tendinopathic Achilles tendons than in healthy tendons (111). Although the exact mechanism of action of NK cells in tendon injury healing is not fully understood, it has been shown that by regulating the activity of NK cells, it is possible to influence the intensity and duration of the inflammatory response, and thus the outcome of tissue repair. For example, MSCs can inhibit the activity of NK cells, thereby exerting an anti-inflammatory effect (112). Therefore, therapeutic strategies targeting NK cells may provide new ideas for the treatment of chronic tendinopathy.

In addition to neutrophils and macrophages, other immune cell lineages such as B cells, T cells, and NK cells have been observed in the tendon during the healing process. While macrophages have received considerable attention in tendon–bone healing research, the functions of these other immune cells remain poorly understood (44, 113). For example, T helper cells have been detected in the tendon 3 days after injury and are believed to promote fibronectin production by migrating fibroblasts (114). The phenotypic heterogeneity and tissue-specific functions of immune cells underscore the complexity of the immune response in tendon–bone healing. Gaining a comprehensive understanding of the involvement of these immune cell lineages is crucial for unraveling the intricate mechanisms underlying tendon–bone healing and for optimizing therapeutic approaches in this context. Further research is necessary to elucidate the contributions of these immune cell lineages and develop targeted therapeutic interventions.

### 3.3 Role of non-inflammatory cells in the inflammatory microenvironment

Non-inflammatory cells, such as fibroblasts and endothelial cells, play a key role in the proliferation and reconstruction phases of tissue healing (115). They are involved in processes such as neovascularization and granulation tissue formation, laying the foundation for subsequent tissue repair (44). Despite the significant role of these cells in the later stages of healing, their specific function in the early microenvironment of inflammation still needs to be thoroughly investigated (116).

#### 3.3.1 Fibroblasts

Fibroblasts play a key role in tendon healing. In addition to their cell proliferation and collagen synthesis functions, fibroblasts are deeply involved in the inflammatory response of tendon tissue (117).

Firstly, fibroblasts are active participants in the critical microenvironment. They express Toll-like receptors (TLRs) that recognize risk- and danger-related molecular patterns, thereby triggering an immune response (118). In addition, fibroblasts activate NF-κB factors in response to epidemic stimuli, further promoting the recruitment of inflammatory cells (119). It has been shown that fibroblasts exhibit sustained activation in injured or inflamed tendon tissue, which is strongly associated with the development of chronic inflammation and tendinopathy (120).

Secondly, fibroblasts play an important role in the regulation of the immune response. They are not only involved in immune cell uptake but also influence the function of immune cells through various cytokines and growth factors. For example, VEGF-A of fibroblasts promotes angiogenesis, providing nutrients and oxygen for tissue repair (121). In addition, fibroblasts are antimicrobial. Their antimicrobial peptides and defense molecules help clear the risk of infection (122).

In summary, fibroblasts not only are involved in tissue reconstruction during tendon healing but also actively regulate the response. They influence the activation and function of immune cells through a variety of mechanisms, thus playing an important role in all stages of tendon healing.

#### 3.3.2 Vascular endothelial cells

Vascular endothelial cells play a crucial role in regulating inflammation during tendon–bone healing. Key aspects of their involvement include the following:

Inflammatory signaling is a crucial aspect of the role played by vascular endothelial cells in tendon–bone healing. Upon stimulation by trauma signals, these cells produce inflammatory cytokines and chemokines, such as interleukins and tumor necrosis factor, initiating an inflammatory response (123). Furthermore, the expression of cell adhesion molecules, including selectins and integrins, by vascular endothelial cells facilitates interactions with circulating inflammatory cells. This expression promotes the capture, rolling, adhesion, and migration of inflammatory cells on the surface of endothelial cells (124, 125). Moreover, vascular endothelial cells exert influence over the infiltration of inflammatory cells by regulating endothelial permeability. Through the opening of intercellular gaps during inflammation, these cells enable inflammatory cells to enter the injured tissue via the interstitium (126). Additionally, vascular endothelial cells release inflammatory mediators, such as chemokines and cytokines, which further modulate the inflammatory response. These mediators attract and activate inflammatory cells, thus promoting tissue repair and regeneration processes (127). In addition to their roles in inflammation, vascular endothelial cells participate in immune modulation by interacting with immune cells. They regulate the intensity and duration of the inflammatory response, playing a significant role in maintaining immune balance and controlling excessive inflammatory reactions (128).

Therefore, by orchestrating inflammatory signaling, expressing adhesion molecules, influencing inflammatory cell infiltration, releasing inflammatory mediators, and participating in immune modulation, vascular endothelial cells play a crucial regulatory role in tendon–bone healing. A comprehensive understanding of their functions and mechanisms in this process can contribute to optimizing treatment strategies, promoting tendon–bone healing, and reducing the occurrence of related complications. Understanding the function and mechanisms of vascular endothelial cells in tendon–bone healing is essential for optimizing treatment strategies to promote healing and reduce associated complications.

In summary, non-inflammatory cells such as fibroblasts and vascular endothelial cells play active roles in the inflammatory phase of tendon–bone healing (**Table 2**). Their secretion of various molecules, expression of TLRs, and involvement in immune cell recruitment contribute to the complex processes of inflammation and tissue repair. Further research is necessary to fully understand the mechanisms underlying their immunomodulatory effects and explore their potential therapeutic applications in the healing of tendon injuries.

**Table 2:** Role of non-inflammatory cells in tendon–bone healing.

Cell Type	Key Role	Mechanisms	Clinical Significance	References
Fibroblasts	Inflammation regulation and tissue repair	TLR signaling, cytokine production, and ECM remodeling	Crucial for tendon healing and potential therapeutic target	(120)
Vascular endothelial cells	Inflammatory signaling, immune cell recruitment, and angiogenesis	Adhesion molecule expression, cytokine release, and permeability regulation	Essential for tissue repair and potential therapeutic target	(126)

TLR, Toll-like receptor; ECM, extracellular matrix.

### 3.4 Role of inflammatory cytokines in the inflammatory microenvironment

After a tendon injury, the inflammatory response makes a natural defense mechanism of the organism. A variety of inflammatory cytokines play a key role in this process (**Table 3**). These cytokines, such as interleukins (IL-6, IL-8, and IL-18), chemokine-like factor 1 (CKLF-1), and prostaglandins (COX-1), not only induce inflammatory responses, but also regulate tissue repair and remodeling, and are frequently elevated in scar tissue associated with tendon injury (135).

**Table 3:** Key inflammatory mediators and their roles in tendon pathology.

Mediator	Primary Function	Role in Tendon Pathology	Specific Manifestations	References
IL-1β	Pro-inflammatory	Induces inflammation, degrades ECM, and inhibits TSPC function	Promotes expression of COX-2, PGE2, and MMP-1; accelerates ECM degradation	(129)
IL-6	Multifunctional	Regulates inflammation, immune response, and collagen synthesis	Promotes collagen synthesis, involved in scar formation, and plays a dual role in inflammation and repair	(130)
IL-10	Anti-inflammatory	Regulates inflammation and inhibits excessive scar formation	Inhibits the production of pro-inflammatory cytokines and promotes tissue repair	(131)
IL-17A	Pro-inflammatory	Induces tissue destruction and inflammation and promotes collagen-type conversion	Promotes the expression of TNF-α, IL-6, and IL-8, and induces apoptosis	(52)
TNF-α	Pro-inflammatory	Promotes inflammation, ECM degradation, and cell apoptosis	Inhibits collagen synthesis and activates the NF-κB signaling pathway	(82)
IL-21	Pro-inflammatory	Induces immune cells and non-immune cells to secrete chemokines and matrix metalloproteinases	Aggravates inflammatory response and tissue damage	(35)
IL-33	Pro-inflammatory/Anti-inflammatory	Induces collagen synthesis, promotes inflammation, and regulates immune response	Promotes collagen synthesis and inflammatory response, and plays different roles at different stages of the disease	(99)
Substance P	Neuropeptide	Mediates pain and causes edema and fibrosis	Activates mast cells and releases inflammatory mediators	(113)
Alarmin molecules (HMGB1, S100A9)	Alarm molecules	Triggers inflammation and promotes ECM remodeling	Promotes inflammatory response and induces collagen synthesis	(132)
CXCL12	Chemokine	Recruits fibroblasts and endothelial cells	Promotes scar formation	(133)
MCP-1	Chemokine	Recruits monocytes and macrophages	Influences inflammation and tissue remodeling	(134)

ECM, extracellular matrix; TSPCs, tendon stem/progenitor cells; COX-2, cyclooxygenase-2; PGE2, prostaglandin E2; MMP-1, matrix metalloproteinase-1; NF-κB, nuclear factor kappa-light-chain-enhancer of activated B cells; HMGB1, high mobility group box 1.

After tendon injury, the body responds mainly through two types of immune responses, type I and type II (98). Necrotic or activated immune cells in type I immune responses release alert elements such as S100A8 and S100A9 into the extracellular environment (68), which triggers the recruitment of immune cells (Th1 T-cells, neutrophils, and M1-type macrophages), and the release of pro-inflammatory factors such as TNF-α, IFN-γ, IL1-β, and iNOS from tendon cells (67). Downstream inflammatory signaling pathways such as NF-κB and NLRP3 are activated to regulate inflammatory genes and promote ECM catabolism and *de novo* deposition (136, 137). To counteract excessive inflammation, a type II immune response is activated, characterized by the secretion of IL-4 or IL-33 by damaged cells. IL-33 triggers a response in macrophages, Tregs, and other immune cells, while IL-4 promotes the conversion of naïve CD4 T cells and macrophages to an anti-inflammatory phenotype (138). Inflammatory factors released during tendon healing (e.g., IL-1 and TNF-α) degrade the ECM, inhibit tendon cell markers, and induce pain (139). IL-1β downregulates the expression of certain genes and promotes the production of matrix-degrading enzymes (129). The binding of IL-1 and TNF-α to the TLR4 receptor initiates a signaling cascade involving multiple proteins and ultimately activates NF-κB, which drives the expression of pro-inflammatory cytokines (140). This pro-inflammatory signaling pathway can be self-perpetuating, leading to a sustained inflammatory response during tendon healing (131). A sustained inflammatory environment can negatively impact tendon healing and lead to the formation of tissue adhesions (119). If there is an imbalance between type I and type II immune responses, this may lead to a pathological state. An over-activated type I immune response may lead to chronic inflammation, delayed healing, and even scar tissue formation (141). In contrast, an insufficient type II immune response may lead to persistent inflammation and compromise tissue repair.

In the early stages of tendon injury, pro-inflammatory cytokines such as IL-1β (142) and TNF-α are released in large quantities, triggering an inflammatory response and removal of necrotic tissue (143). As the injury is repaired, anti-inflammatory cytokines gradually take over, inhibiting the inflammatory response and promoting tissue regeneration. Anti-inflammatory cytokines such as IL-10 inhibit macrophage activation and reduce the production of pro-inflammatory cytokines and, at the same time, promote macrophage polarization towards the M2 type, secrete growth factors, and stimulate fibroblast proliferation and collagen synthesis (144).

An imbalance in cytokine action at any stage of tendon healing can lead to abnormal scar formation (98). Understanding the complex role of cytokines in tendon pathology can help to provide insight into the underlying mechanisms of inflammation, tissue remodeling, and scar formation, and help to identify potential therapeutic targets to promote normal tendon healing, prevent pathological scarring, and improve patient prognosis.

### 3.5 Role of signaling pathways in the inflammatory response in tendon–bone healing

Key signaling pathways involved in tendon–bone healing include NF-κB, NLRP3, p38/MAPK, and STAT3 (**Table 4**). These pathways regulate inflammatory factor expression, influencing inflammation, cell proliferation, and tissue remodeling (146). Excessive inflammation can hinder tendon healing and lead to adhesions (53). Understanding the specific mechanisms of these signaling pathways is crucial for developing strategies to optimize tendon healing and reduce inflammation. By targeting these pathways, we can potentially improve treatment outcomes for tendon–bone injuries.

**Table 4:** Key signaling pathways in tendon–bone healing.

Signaling Pathway	Role in Tendon Healing	Key Mediators	Therapeutic Implications	References
NF-κB	Regulates inflammation, cell proliferation, and ECM remodeling	IL-1β, IL-6, TNF-α, CCL2, and CXCL10	Targeting NF-κB may reduce inflammation and promote healing	(119)
NLRP3 Inflammasome	Mediates inflammatory response	HMGB1, IL-1β, and IL-18	Inhibiting NLRP3 may reduce inflammation and improve healing	(132)
p38/MAPK	Regulates inflammation, cell growth, and apoptosis	TNF-α, IL-6, and IL-8	Targeting p38/MAPK may attenuate inflammation and reduce heterotopic ossification	(145)
STAT3	Regulates inflammation, cell proliferation, and differentiation	IL-10 and TIMP-3	Targeting STAT3 may modulate inflammation and ECM remodeling	(33)

NF-κB, nuclear factor kappa-light-chain-enhancer of activated B cells; NLRP3, NOD-like receptor family pyrin domain containing 3; MAPK, mitogen-activated protein kinase; STAT3, signal transducer and activator of transcription 3; IL-1β, interleukin-1 beta; IL-6, interleukin-6; TNF-α, tumor necrosis factor-alpha; CCL2, C-C motif chemokine ligand 2; CXCL10, C-X-C motif chemokine ligand 10; ECM, extracellular matrix; HMGB1, high mobility group box 1; TIMP-3, tissue inhibitor of metalloproteinases-3.

#### 3.5.1 Role of the NF-κB signaling pathway

NF-κB, a pivotal transcription factor governing inflammation, manifests as a heterodimer (p50/p60) post-tendon injury, instigating the release of inflammatory mediators like IL-1β, IL-6, TNF-α, CCL2, and CXCL10 (147). It orchestrates multiple facets of tendon healing—from inflammation to cell proliferation, angiogenesis, and tissue adhesion formation (148). In the inflammatory phase, NF-κB acts as a pro-inflammatory signaling pathway, chiefly modulating the expression of pro-inflammatory genes and cytokines (149). Immune cells such as neutrophils and macrophages release inflammatory factors like IL-1β and TNF-α post-tendon injury (139). The canonical NF-κB activation route involves ligand-receptor binding for TNF or IL-1, alongside pattern recognition and antigen receptors, with resident tendon fibroblasts housing these receptors. In tendinopathy, there is an upsurge in corresponding cytokine expression (144, 150, 151). Recently, in early rotator cuff tendinopathy, interleukin-33 (IL-33) was found to spur canonical NF-κB signaling via ST2, suppressing micro-RNA-29a in tendon cells and prompting type III collagen production for scar formation. NF-κB activation unfolds through the canonical pathway involving IκB phosphorylation and degradation, alongside the non-classical pathway spurred by CD40 and hypoxia (152–154).

Selective inhibition of NF-κB activity, especially via IKKβ inhibition, can dampen the inflammatory response (155). A canine study on tendon repair showcased that oral ACHP (an IKKβ inhibitor) administration diminished phosphorylated p65 (p-p65) levels by day 14, curbing inflammation-related gene expression and boosting cell proliferation and neovascularization, expediting healing (156). Likewise, administering ACHP in a rat rotator cuff injury model demonstrated reduced inflammation-related gene expression in the NF-κB pathway and enhanced ECM production at the injury site. *In vitro* experiments with stimulated rat tendon fibroblasts yielded promising results (157). NF-κB-induced cytokine-stimulated fibroblasts proliferate, synthesize collagen fibers, and transition into myofibroblasts, fostering scar tissue formation (131, 147). Elevated p65 expression in adhesion tissues implies NF-κB signaling’s ties to fibrotic adhesion formation in tendon repair (158).

NF-κB signaling cascades influence IL-6 production, a cytokine with dual roles. While traditionally viewed as proinflammatory for T-cell activation, macrophage activation, and osteoclast formation, IL-6 now emerges as an anti-inflammatory myokine, responding to physical exercise by aiding inhibitory factor secretion like IL-1ra and IL-10 (159). IL-6’s pro-inflammatory tendencies are modulated by TNF-α (160). In patients with RCTs, IL-6 gene expression and cytokine production correlate with degeneration extent and genes linked to tissue remodeling (161–164). Studies in rotator cuff repair patients have linked increased joint stiffness to IL-6 gene polymorphisms (130). Exploration into inhibiting adhesion formation via IL-10 or hyaluronic acid injections is ongoing, necessitating further research to elucidate molecular mechanisms and refine clinical applications (165).

#### 3.5.2 Role of the NLRP3 signaling pathway

The inflammasome, a signaling complex comprising multiple proteins recognizing pathogen-associated molecular patterns (PAMPs) and damage-associated molecular patterns (DAMPs), plays a crucial role in various diseases (166). The NLRP3 inflammasome, a common complex in tendinopathy inflammation, consists of the NLRP3 sensor, ASC adapter, and pro-caspase1 (167). Activation of NLRP3 by diverse signals, both exogenous and endogenous, leads to cytokine release, intensifying the inflammatory response (133). Following tendon injury, HMGB1 release from necrotic cells is a primary pathway for NLRP3 activation, stimulating the assembly and activation of the NLRP3 inflammasome (137). Activated NLRP3 triggers cytokine release, disrupts the ECM, and amplifies inflammation, impeding tendon healing. Various cellular signals, such as K+ efflux, Ca2+ signaling, and mitochondrial dysfunction, can activate the NLRP3 inflammasome, necessitating further investigation into their specific roles (168).

HMGB1 binds to cell membrane receptors (RAGE, TLR2, TLR4, and TREM-1), activating NLRP3, which assembles apoptosis-associated speckle-like protein (ASC) and recruits caspase-1 for processing (169). Active caspase-1 converts pro-IL-1β and pro-IL-18 into their mature, biologically active forms. Elevated Ca2+ levels disrupt F-actin function, releasing NLRP3 inhibition and enhancing IL-1β maturation and release (170).

Persistent adipose tissue infiltration in tenocytes prolongs inflammation and hampers tendon healing (171). Studies in hepatocytes suggest a synergistic effect between TLR2, TLR4, TREM-1, and RAGE in upregulating HMGB1 and promoting adipose tissue infiltration (172). Inhibition of NLRP3 activity prevents adipose tissue accumulation in hepatocytes, hinting at a potential role of NLRP3 in adipose tissue infiltration across tissues (173). Ion fluxes, including K+, Ca2+, and Cl−, activate the NLRP3 inflammasome. F-actin interacts with calcium-dependent proteins, inhibiting NLRP3 activation. Cyclic stretching of tenocytes increases Ca2+ levels, depolymerizing F-actin and relieving NLRP3 inhibition, leading to heightened IL-1β release (174).

#### 3.5.3 Role of the p38/MAPK signaling pathway

MAPKs, critical protein kinases involved in phosphorylation, transmit extracellular signals within organisms (175). In tendinopathy, MAPKs regulate inflammation, ECM dynamics, and apoptosis (176). The p38/MAPK pathway, activated under stress, governs inflammation, cell growth, and apoptosis (177). Activation involves multiple kinase phosphorylations for signaling transmission (145). GTPases activate MAPKKK, which then activates MAPKK (MKK3 and MKK6 in p38 pathway), phosphorylating MAPK and inducing inflammatory factor production like TNF-α, IL-6, and IL-8 to regulate inflammation (175).

Enhanced NF-κB and p38/MAPK activity prompts inflammation and heterotopic ossification (HO) (178). *In vitro* studies using MAPK inhibitors (e.g., SB203580, PD98059, and SP600125) showed reduced phosphorylation of ERK, JNK, p38, NF-κB, and IKKβ, along with decreased HO, suggesting MAPK inhibition as a therapeutic strategy for attenuating inflammation, warranting further human *in vivo* research (179).

#### 3.5.4 Role of the STAT3 signaling pathway

The STAT pathway is a vital signaling pathway comprising STAT proteins and their receptors. Within the STAT protein family, STAT3 stands out as a significant member, contributing to immune response, cell proliferation and differentiation, angiogenesis, apoptosis, and tumorigenesis (180). Various chemical signals such as interferons, interleukins, and growth factors bind to cell surface receptors, initiating a cascade of events involving associated kinases. These kinases phosphorylate themselves, the receptor, and downstream target proteins, including the transcription factor STAT3. Phosphorylated STAT3 forms dimers that translocate to the nucleus, binding to target genes and regulating downstream gene transcription (181).

In the context of the tendon’s inflammatory response, STAT3 displays dual regulatory roles, although the exact mechanisms remain unclear. On one hand, inhibiting JNK/STAT3 signaling has been shown to significantly increase the expression of the proinflammatory factor IL-6, which promotes wound healing through fibrosis and scarring (33). However, persistent inflammation often leads to excessive myofibroblast proliferation and the excessive deposition of ECM components, resulting in scar tissue formation (182). On the other hand, the STAT signaling pathway is essential for IL-10 to exert its anti-inflammatory effects and stimulate IL-10 production (33). For instance, IL-10 regulates the TLR4/NF-κB signaling axis in dermal fibroblasts via the IL-10R/STAT3 pathway, effectively reducing the inflammatory response and preventing scar formation (165).

IL-10 forms honor heterodimers by binding to the IL-10R receptor, activating the receptor-linked tyrosine-protein kinase 1 (JAK1) (183). Stimulation of tendon stem cells (TSCs) with connective tissue growth factor induces the production of the anti-inflammatory cytokine IL-10 through the JNK/STAT3 signaling pathway, effectively reducing inflammation, ECM secretion, and scar tissue (184). Additionally, the JNK/STAT3 signaling pathway plays a crucial role in aspirin-induced expression of IL-10 and TIMP-3 in TSCs. Aspirin upregulates the expression of the anti-inflammatory factor IL-10 via the JNK/STAT3 signaling axis, inhibiting the migration and proliferation of TSCs induced by IL-1β. Consequently, the reduction in TSCs may decrease ECM deposition in the injured tendon, ultimately reducing scar formation. As such, aspirin indirectly improves tendon healing by regulating inflammation through the JNK/STAT3 signaling pathway (33). Besides pharmacological interventions, recent research has explored the use of mechanical stimulation to modulate the inflammatory response. For example, mechanical stimulation of tendons through treadmill running increases the expression of IL-4 at the tendon–bone junction. The binding of IL-4 to its receptor activates JAK1 and JAK3, leading to the activation of STAT and its translocation to the nucleus. Ultimately, the JAK/STAT signaling pathway shifts macrophages from a pro-inflammatory to an anti-inflammatory phenotype, resulting in the production of various anti-inflammatory cytokines that regulate the local inflammatory microenvironment and promote tissue healing (185).

However, further in-depth studies, including sequencing analysis, are necessary in the future to elucidate the specific mechanisms and targets involved in the overall inflammatory regulatory process. Therefore, STAT3 plays a crucial role in maintaining the balance between inflammation and ECM during the healing of injured tendons.

### 3.6 Role of mechanical load in the inflammatory response

Mechanical loading plays a significant role in influencing the metabolism and healing process of tendons (186). Tendons, being responsive to mechanical forces, exhibit remarkable mechanosensing capabilities. They are sensitive to various types, frequencies, and amplitudes of loading, which, in turn, impact their metabolic activities and regenerative potential (187).

One crucial aspect influenced by mechanical loading is tendon metabolism. The application of mechanical pressure on tendons has been found to modulate the synthesis and breakdown pathways, thereby affecting their overall metabolic processes (188). It has been observed that mechanical loading can regulate inflammation and immune responses within tendons (189). This regulation involves the upregulation of both pro-inflammatory and anti-inflammatory factors, leading to a complex interplay between inflammatory cells and tendon cells (190). In animal experiments, the loading of tendons has shown interesting effects on the early inflammatory response and tissue regeneration. The loading-induced elongation of early inflammation has been observed, along with an increase in tendon cross-sectional area (191). Moreover, mechanical loading has been found to polarize macrophages, with a higher prevalence of M1 macrophages over M2 macrophages (192). Additionally, during the regenerative phase, a delayed inflammation characterized by an increased number of M2 macrophages and Tregs has been observed. The influence of mechanical loading extends beyond inflammation and immune responses. Tendon cells respond to mechanical stimuli by activating intricate cell signaling pathways. These pathways, in turn, affect the synthesis, breakdown, and homeostatic activities in the ECM (192). Collagen bundles, the main force transmitters to tendon cells, primarily transmit uniaxial tension. On the other hand, larger collagen fibers and ECM structures transmit lesser shear and compressive forces during loading (193). Once a certain load threshold is reached, tendon cells initiate complex cellular responses, potentially involving the activation of inflammation-related pathways (194).

Mechanical loading significantly affects tendon metabolism and healing, influencing inflammation, immune responses, macrophage polarization, and regenerative phase inflammation (195). Tendon cells’ mechanosensing capacities and responses to various loads, frequencies, and amplitudes, coupled with ECM interactions and cell signaling pathways, determine the balance between synthesis, breakdown, and homeostasis (196). Unveiling these mechanisms enhances our understanding of tendon biology, paving the way for innovative approaches to managing tendon-related conditions.

## 4 Reagents used in immune regulation

The modulation of inflammatory responses and macrophage polarization during the healing process of tendon–bone injuries can be achieved through the use of small molecules (197). These molecules can be administered through tissue engineering approaches or systemic delivery and have shown promising results in regulating the healing outcome.

One effective strategy involves the use of electrospun membranes made of polycaprolactone (PCL) and melatonin (90). These membranes exhibit immunomodulatory effects and have been found to enhance the healing of rat supraspinatus tendon–bone injuries. The controlled release of melatonin from the electrospun membranes reduces macrophage infiltration during the tendon–bone healing process, promoting the formation of fibrocartilage and reducing fibrovascular tissue formation. Another approach utilizes a fibroin gel that delivers acetylcholine and pyridostigmine, both of which possess anti-inflammatory properties (198). When applied to the tendon–bone interface in a mouse model of rotator cuff injury, this gel promotes the regeneration of fibrocartilage-like tissue during the healing phase. Magnesium-preconditioned bone membranes have also shown promising results in promoting tendon–bone healing (199). In a rabbit ACL reconstruction model, bone membranes pre-treated with magnesium enhance the polarization of M2 macrophages surrounding the tendon–bone interface. This polarization effect prevents bone loss around the tunnel and increases the maximum failure load of the graft. Chondroitin sulfate and bone morphogenetic protein-2 (BMP-2) attached to polyethylene terephthalate (PET) ligaments have been demonstrated to promote tendon–bone healing and bone regeneration (200). The presence of chondroitin sulfate on PET ligaments increases the expression of Arg-1 and works synergistically with BMP-2 to facilitate bone regeneration during the tendon–bone healing process in a rat proximal tibia transplantation model (197). Furthermore, dithiothreitol has been found to reduce the release of pro-inflammatory cytokines, such as IL-1β and TNF-α, while promoting the polarization of M1 macrophages towards the M2 phenotype. This molecule reduces macrophage infiltration, inhibits tendon fibrosis, and promotes fibrocartilage and bone regeneration during the healing process in a mouse model of Achilles tendon injury (201).

The use of small molecules with immunomodulatory properties to regulate macrophage polarization during tendon–bone healing holds great promise. By modulating the inflammatory response and promoting the polarization of macrophages towards the M2 phenotype, these molecules have the potential to enhance the healing outcome and facilitate tissue regeneration.

### 4.1 Steroids

Steroids are widely used for their anti-inflammatory properties in the treatment of autoimmune diseases and scar reduction (202). In addition, oxymetholone, a hormone commonly used for treating wasting diseases, has recently been found to have anti-fibrotic effects, offering potential benefits in scar treatment (203).

When it comes to scar treatment, steroids have gained recognition for their anti-inflammatory properties. This makes them effective in treating autoimmune diseases and reducing scar formation (204). One successful method of delivering hormones is through intradermal injection, such as using triamcinolone (205). This approach has shown high success rates in scar treatment by suppressing the activity of bone marrow and lymphocytes. As a result, inflammation is reduced, and the appearance of scars improves (206). However, caution should be exercised when using steroids for painful tendon diseases. Hormone delivery to tendons can lead to acute detrimental changes in healthy tendons, including collagen necrosis, collagen disarray, and infiltration of inflammatory cells (207). Overall, the mechanical properties of healthy tendons may be negatively affected by steroid administration (208). Therefore, it is essential to use steroids cautiously in the treatment of painful tendon conditions, carefully considering the potential risks and benefits for each patient. On the other hand, oxymetholone, which is traditionally used for treating wasting diseases, has shown promise in reducing fibrosis and scar formation (209). It exhibits beneficial effects by decreasing wound inflammation, inhibiting fibroblast and myofibroblast activity, and preventing excessive collagen deposition (210). These anti-fibrotic effects of oxymetholone offer potential benefits for scar treatment and may contribute to improved wound healing outcomes.

In conclusion, steroids play a valuable role in scar treatment due to their anti-inflammatory properties. However, it is crucial to exercise caution when using them for painful tendon conditions. Nonetheless, further research is necessary to fully understand the benefits and risks associated with these treatments.

### 4.2 NSAIDs

NSAIDs, such as aspirin, are widely utilized for their anti-inflammatory properties, which stem from their ability to inhibit the production of prostaglandins, thus alleviating inflammation (211). In addition to their primary function, these drugs have attracted attention for their potential anti-fibrotic effects and their role in promoting the healing of tendons (209).

Studies have demonstrated that aspirin exhibits anti-fibrotic properties by inhibiting the activation of NF-kB (53) induced by TNF-α, thereby impeding fibroblast proliferation (212). Moreover, research conducted by Wang et al. has shown that aspirin facilitates the differentiation of TSCs into tenocytes and contributes to tendon healing. This process has been associated with the GDF7/Smad1/5 signaling pathway (213). Furthermore, *in vivo* experiments have observed that aspirin limits inflammation and suppresses post-injury tendon scar formation by modulating the JNK/STAT-3 signaling pathway (33). On the other hand, aspirin has been found to decrease the ratio of tissue inhibitor of metalloproteinase-3 (TIMP-3) to matrix metalloproteinase-3 (MMP-3), resulting in ECM degradation (33). However, it is worth noting that while NSAIDs, including aspirin, block prostaglandin production derived from arachidonic acid, leading to an overall decrease in inflammation, excessive arachidonic acid can also cause tissue damage (214). Therefore, aspirin not only possesses anti-inflammatory and pain-relieving properties but also holds the potential for the treatment of tendon diseases. Nonetheless, the long-term use of aspirin may have detrimental effects on tissue and tendon healing processes.

It is important to emphasize that a transient inflammatory phase is necessary for proper tendon healing. Studies using rat models have shown that administering common non-steroidal anti-inflammatory drugs (NSAIDs) immediately after a patellar tendon or rotator cuff injury and repair can reduce tendon mechanical properties, indicating that suppressing early inflammation can significantly affect the subsequent healing response (157, 215, 216).

In summary, NSAIDs, particularly aspirin, are widely utilized for their anti-inflammatory properties, but their prolonged use should be approached with caution due to potential adverse effects on tissue and tendon healing. *In vitro* studies have also demonstrated that exposing tenocytes to NSAIDs decreases their rate of proliferation and the production of matrix proteins (157, 217). The emerging evidence of their anti-fibrotic effects and their role in tendon healing offers promising avenues for further research and potential therapeutic applications in the field.

### 4.3 Signaling inhibitor

In the realm of treatment strategies, a targeted and precise method involves focusing on signaling inhibitors to address specific immune pathways, such as the NF-κB pathway, known for its pivotal role in regulating inflammatory responses (218). The activation of pro-inflammatory mediators via the NF-κB pathway, typically triggered by TNF and IL-1 signaling cascades, stands out as a critical factor in inflammation modulation (219). Consequently, researchers have honed in on manipulating these pathways to either treat diseases effectively or enhance healing processes.

For instance, TNF inhibition has proven successful in managing autoimmune conditions like rheumatoid arthritis and ankylosing spondylitis (220). In contrast, utilizing low-dose inhibition has shown promise in ameliorating tendon diseases without heightening infection risks. In a preliminary study involving symptomatic athletes with Achilles tendon issues, administering the TNF-α monoclonal antibody, adalimumab led to reduced walking and resting pain, maintained tendon thickness, and reduced blood flow over a 12-week treatment period (221). Similarly, in a rat model focusing on rotator cuff repair, blocking TNF-α using a polyethylene glycosylated TNF receptor type 1 bolstered biomechanical strength and reduced pro-inflammatory macrophages in early stages (within 4 weeks) (82). However, these effects normalized compared to untreated controls at 8 weeks. In a separate rat model of adhesive capsulitis induced by carrageenan, silencing TNF-α via lentiviral-mediated siRNA at 5 weeks tempered the inflammatory response and decreased fibrocartilaginous metaplasia (222).

Inhibiting the IL-1 signaling pathway has also shown efficacy in treating autoimmune diseases and presents a potential therapeutic avenue for shoulder conditions, including shoulder tendon diseases (223). In the athlete study mentioned earlier, administering anakinra (a recombinant IL-1 receptor antagonist) for Achilles tendon problems yielded less significant results compared to TNF inhibitors (146). Recent animal studies, however, have displayed more promising outcomes for IL-1 inhibition. For example, in rabbit and rat models of tendon issues, IL-1ra treatment prevented mechanical degradation, maintained collagen integrity, and reduced inflammatory cell infiltration (224).

Moreover, exploring the inhibition of matrix metalloproteinases (MMPs), crucial enzymes in ECM degradation downstream of inflammatory responses, has shown promise in treating musculoskeletal degenerative diseases (225). While doxycycline, an MMP inhibitor, improved healing following rotator cuff repair in rats, its effects on rat Achilles tendons have yielded mixed results (226). Another inhibitor, recombinant a-2-macroglobulin, demonstrated potential in enhancing tendon regeneration and improving bone interface after acute rotator cuff repair in rabbits (227).

In conclusion, targeting immune signaling pathways through signaling inhibitors holds significant promise in promoting tissue healing, particularly in bone repair. By regulating specific immune signaling cascades like NF-κB, TNF, and IL-1, it becomes possible to modulate inflammatory responses effectively, thereby aiding in tissue repair processes (136). While TNF inhibitors have shown success in autoimmune disease management and low-dose inhibition presents hope for tendon conditions (228), IL-1 inhibitors are emerging as a potential therapeutic option for shoulder ailments (229). Additionally, delving into MMP regulation offers a novel avenue for addressing musculoskeletal degenerative diseases (230). However, further comprehensive studies are necessary to fully grasp the therapeutic potential and applicability of these approaches.

## 5 Mesenchymal stem cell-mediated modulation of inflammation

Rotator cuff degeneration and tendon ailments pose a challenge due to their intricate inflammatory and tissue repair processes. However, exploring diverse treatment approaches with distinct mechanisms has unveiled potential solutions. MSCs offer hope for enhancing tendon healing by regulating the inflammatory milieu (231). Specifically, adipose-derived stem cells (ASCs) have exhibited protective effects on local cells through their secretion of soluble factors (232).

Co-culture investigations have successfully shown that ASCs can counteract the impact of pro-inflammatory macrophages on tendon fibroblasts, inducing a shift from a pro-inflammatory to an anti-inflammatory macrophage phenotype. This shift reduces the exposure of tendon fibroblasts to pro-inflammatory cytokines like IL-1b and boosts the production of ECM proteins (233). Moreover, in animal models, ASCs administered at the tendon repair site have demonstrated the capacity to modulate the inflammatory response and enhance healing outcomes (234). These results, backed by gene expression and immunohistochemical analyses, underscore the potential of ASCs in improving tendon healing by adeptly managing inflammation.

Furthermore, studies involving MSCs stimulated with the cytokine TNF-α have reported similar positive outcomes (235). Notably, treating canine flexor tendons with autologous ASCs led to an increase in M2 macrophages, higher levels of M2-activated IL-4, and a decrease in scar-related gene expression (236). Collectively, these findings emphasize the promising role of MSCs and ASCs in optimizing tendon healing by effectively regulating inflammation.

## 6 Exosomes enhance tendon–bone healing

Recently, exosomes secreted by various cells have paved the way for novel therapeutic approaches (237). Their anti-inflammatory, anti-oxidative stress, anti-fibrotic, and pro-angiogenic properties make them important in promoting tissue regeneration (238). Exosomes facilitate intercellular communication by transferring various molecules, including proteins, lipids, and genetic materials such as messenger RNA, microRNA, and other non-coding RNAs (239, 240), to regulate cellular processes in both physiological and pathological states (241, 242). Notably, numerous studies have found that exosomes derived from MSCs serve as crucial regulatory factors in the microenvironment of tendon–bone healing, playing a significant role in the healing process of tendon–bone injuries (243–247). Thus, cell-free therapies based on exosomes may provide safer and more effective treatments for tendon disorders, offering researchers a new perspective on promoting tendon–bone healing.

### 6.1 The role of exosomes in modulating immune and inflammatory responses

Exosomes hold promise for treating tendon injuries by modulating the inflammatory response. Studies have shown that MSC-Exos can attenuate inflammation, reduce scar tissue formation, and promote tissue regeneration (248, 249). This is likely due to their ability to regulate immune cell function and influence the microenvironment surrounding injured tendons. Early and robust inflammatory responses during tendon healing can lead to the formation of scar tissue in the later stages (132). Subsequent to scar formation, tendons undergo histological, biochemical, and biomechanical changes, resulting in compromised strength and elasticity, making them susceptible to re-injury (250). Therefore, effective control of inflammation following tendon injury is crucial for promoting high-quality healing.

The administration of ASC-Exos, derived from adipose-derived stem cells, in the treatment of tendons affected by tendinopathy, was shown by Wang et al. to significantly improve the inflammatory state, histological characteristics, and biomechanical strength. Tendinopathic tendons treated with ASC-Exos exhibited reduced cellularity, angiogenesis, ground substances, and collagen fiber disruption compared to tendinopathic tendons treated with saline. The key factor underlying these therapeutic effects was identified as the enhanced polarization of M2 macrophages, highlighting the role of exosomes in promoting tissue healing (251). Ye et al. investigated the efficacy of iMSC-lEVs [extracellular vesicles (EVs) derived from induced MSCs] in improving pain-related behaviors in a tendinopathy rat model (201). Immunohistochemical analysis revealed that iMSC-lEVs reduced the expression of inflammatory markers (IL-1β, TNF-α, IL-6, and NGF) in rat tendon tissues. These findings demonstrate that iMSC-lEVs effectively alleviate pain in tendon injury, which is closely associated with the amelioration of the inflammatory microenvironment. Excitingly, a notable study conducted by Jenner et al. demonstrated a significant decrease in bony spur formation at the infraspinatus enthesis following the administration of hUC-MSC-derived EVs (sEVs) (252). This finding suggests the potential of utilizing these sEVs to mitigate osteophyte formation associated with injury and inflammation (253).

Overall, these studies suggest that exosomes enhance tendon healing through immunomodulatory effects and hold great promise in the treatment of tendon injuries (**Table 5**).

**Table 5:** Exosome-mediated immunoregulation of enhanced tendon–bone healing.

Animal Model	Source of Exosomes	Results	The Underlying Mechanisms	References
Rat model of the anterior cruciate ligament construction	Infrapatellar fat pad (IPFP) MSC-derived exosomes	IPFP MSC-derived exosomes accelerated tendon–bone healing and intra-articular graft remodeling after ACLR	Decreased M1 expression and increased M2 expression by the immunomodulation of macrophage polarization	(254)
Mice model of Achilles tendon–bone reconstruction	Mice BMSC-derived exosomes	The biomechanical properties of the tendon–bone junction were significantly promoted in the hydrogel BMSC-Exos group	Decreased the M1 macrophages, the proinflammatory factors (IL-1b and IL-6) in local tissues and cell apoptosis, increased cell proliferation, reduced ECM deposition, and suppressed excessive scar formation by regulation of the transition of macrophages from M1 to M2	(255)
Rat model of aged-chronic rotator cuff tear	Healthy tendon stem cell-derived exosomes	Changing the microenvironment at the tendon–bone interface from pro-inflammatory to anti-inflammatory in the acute postoperative period and improving tendon–bone healing	Polarizes macrophages from M1 phenotype to M2 phenotype	(256)
Rabbit model of chronic rotator cuff tear	Human ASC-derived exosomes	Local injection of ASC-Exos in chronic RCTs at the time of repair could prevent the progress of fatty infiltration, promote tendon–bone healing, and improve biomechanical properties	Reduced infiltration of inflammatory cells (e.g., macrophages) into the tendon–bone junction, thus reducing fibrous scar tissue formation	(251)
Ovine model of acute rotator cuff tear	Human umbilical cord (hUC) MSC-derived exosomes	Improved orientation of collagen fibers and less osteophyte formation at the injury site. The fibrocartilaginous transition zone was formed, inflammation at the lesion site was alleviated and fibrotic adhesions were significantly reduced	Inhibited the proliferation of CD3/CD28 stimulated T cells	(252)
Rat model of quadriceps tendinopathy	Human IP-MSC exosomes	Alleviated acute pain in tendinopathy as well as inhibiting activated mast cell infiltration and interactions with nerve fibers	Relieved tendinopathy-related pain through inhibiting mast cell activation via the HIF-1 signaling pathway	(104)

ACLR, anterior cruciate ligament reconstruction; ASC, adipose stem cells; BMSC, bone marrow stromal cells; ECM, extracellular matrix; IP-MSC, induced pluripotent mesenchymal stem cells; MSC, mesenchymal stem cells.

### 6.2 The role of exosomes in promoting angiogenesis

Limited vascularization is a characteristic of tendons, particularly at the tendon–bone junction (TBI), in contrast to muscles. Following a TBI injury, this inadequate vascular supply results in decreased oxygen, growth factor, and nutrient availability crucial for tendon–bone healing. Consequently, this deficiency negatively impacts the biomechanical properties of the tendon and ultimately hampers the process of tendon–bone healing.

Numerous studies have highlighted the critical role of blood supply in tendon–bone healing, emphasizing that revascularization of the tendon contributes to improved healing outcomes (257, 258). Demirag et al. demonstrated that a higher degree of vascularization leads to a more mature histomorphology of the tendon–bone interface, characterized by an increased presence of Sharpey’s fibers (81). Yoshikawa et al. observed that the absence of graft revascularization in the early stages of ACL reconstruction resulted in surgical failure when the angiogenesis-triggering mechanism was lacking (259). Takayama et al. reported that inhibiting the expression of VEGF at the tendon–bone interface impeded angiogenesis, reduced tendon maturity, and compromised biomechanical strength (260). Conversely, their study also revealed that overexpression of VEGF hindered the improvement of grafted tendon strength. While some studies have suggested a detrimental effect of vascular growth on tendon–bone healing, the majority of these studies actually concluded that vascular growth is associated with degenerative tendinopathy (261). Fealy et al. noted an initial rise and subsequent decline in blood supply after rotator cuff reconstruction. They highlighted the superior blood supply surrounding the tendon compared to the poorer supply at the fixation point or bone groove, suggesting that improving blood flow at these sites could enhance tendon–bone healing quality (262).

Huang et al. conducted *in vitro* studies demonstrating that BMSC-Exos (EVs derived from bone marrow MSCs) enhance the proliferation, migration, and formation of angiogenic tubes in human umbilical vein endothelial cells (HUVECs) (263). They also observed that BMSC-Exos activates both the VEGF and Hippo signaling pathways (264). Specifically, the activation of the Hippo signaling pathway by BMSC-Exos does not solely rely on the VEGF signaling pathway. This implies that BMSC-Exos possesses broad and active effects in promoting angiogenesis. Furthermore, their subsequent *in vivo* experiments demonstrated that BMSC-Exos effectively stimulates angiogenesis in the vicinity of the tendon–bone interface. Consequently, further studies on the mechanisms by which exosomes promote angiogenesis via the VEGF signaling pathway and the Hippo signaling pathway may provide avenues for enhancing tendon–bone healing.

## 7 Platelet-rich plasma technologies

Platelet-rich plasma (PRP) is a blood product that promotes tissue repair. PRP contains a complex mixture of growth factors and other bioactive molecules that stimulate cell growth, differentiation, and migration. Clinical studies have shown PRP to be effective in treating tendon and ligament injuries by promoting tissue regeneration and reducing inflammation.

Anitua et al. observed that injecting PRP into sheep tendons led to organized tissue structure and increased cell density, while the control group displayed chaotic cell aggregation (265). PRP also induced vascular changes. Lyras et al. found in a rabbit model that PRP accelerated tendon healing, promoted new blood vessel formation, and improved scar tissue quality (266). Studies on the HMGB1 protein released by platelets, including research by Zhang et al., highlighted its enhancing effect on PRP in tendon healing (267). PRP can reduce pro-inflammatory macrophages and regulate inflammation, potentially improving tendon repair (268). Inflammation plays a crucial role in tendon regeneration responses, with HMGB1 aiding in cell migration and proliferation, further enhancing tendon healing.

In essence, PRP, rich in platelets, stimulates tendon cell functions, angiogenesis, and tissue remodeling through growth factors and cell factors. While PRP shows promise in treating tendon injuries, refining treatment protocols and validation through more research and clinical trials are essential.

## 8 Discussion

This review emphasizes the importance of understanding the role of inflammation in tendon–bone healing and discusses potential therapeutic strategies for optimizing healing outcomes. Tendon injuries pose significant challenges in clinical settings due to the complex structural, compositional, cellular, and biomechanical aspects involved in tendon healing. Inflammation plays a crucial role in the tendon healing process. Within the inflammatory microenvironment, inflammation orchestrated by cytokines and immune cells facilitates debris clearance, tendon cell proliferation, and collagen fiber generation. However, excessive inflammation can lead to tissue damage and tendon adhesion, hinder tendon healing, and promote scar formation. Therefore, achieving a delicate balance between beneficial and detrimental aspects of the inflammatory response is essential for optimal healing outcomes.

To gain a better understanding of the role of inflammation in tendon–bone healing, this review provides an overview of its function and discusses potential mechanisms. It highlights the importance of understanding the interactions between different cell types and the ECM. The article also explores the roles of fibroblasts, inflammatory cytokines, chemokines, and growth factors in promoting healing, inhibiting scar formation, and facilitating tissue regeneration. Macrophages play a significant role in mediating inflammation, cell proliferation, tissue deposition, scar formation, and remodeling. The role of macrophages is multifaceted and varies considerably based on the functional phenotype of the cells and the stage of tendon healing. Studies attempting to inhibit or enhance macrophage activity have provided valuable insights into the role of macrophages in tendon healing, but further information is needed for clinical translation. The use of transgenic or genetically modified animals will continue to enhance our understanding of macrophage function and the cellular and molecular mechanisms of tendon healing. Further research in these areas may provide new insights for the development of targeted therapies for tendon injuries.

The article also discusses the mechanistic roles of several major signaling pathways associated with tendon inflammation and summarizes the main pathways through which inflammation occurs, providing new perspectives for inhibiting ongoing inflammatory responses and treating tendon diseases. The inflammatory response plays a crucial role in effective tendon repair. Initiating the inflammatory response promotes the release of cytokines and growth factors, facilitates the clearance of necrotic substances and ECM fragments, and aids in the smooth progression of the proliferation and remodeling stages of the healing process. However, sustained inflammatory responses can also have negative effects. If the early inflammatory response to tendon repair persists unabated, excessive recruitment of pro-inflammatory mediators occurs. In particular, transcription of pro-inflammatory mediators induced by the NF-κB signaling pathway is considered a key regulatory factor in the inflammatory response. Prolonged inflammatory environments lead to increased migration and generation of tendon fibroblasts, excessive collagen synthesis, and ECM over-deposition, resulting in the formation of dense fibrotic adhesion structures, which are important factors in the subsequent development of chronic degenerative tendon diseases.

Current research indicates that the specific inhibition of inflammatory pathways using inhibitors can significantly suppress the activity of these pathways and alleviate the inflammatory response. However, there are still limitations in their current application. Clinical trials have used a limited number of human cases, and there are no clear standards for the dosage and duration of administration of pathway-specific inhibitors, necessitating further experimentation. Additionally, many small-molecule inhibitors are systemically delivered, which may not be an ideal treatment approach, thus requiring further exploration of drug delivery modalities. For some natural compounds with similar effects as inhibitors, although phase I clinical trials have shown no adverse reactions in human subjects at high doses, further research at the cellular and molecular levels is needed to ensure their safety and efficacy.

In recent years, extensive research has been conducted on the inflammatory pathways associated with tendon diseases, revealing significant potential for targeted anti-inflammatory treatments. Therefore, gaining a comprehensive understanding of the signaling pathways involved in inflammatory responses is crucial for the timely elimination of detrimental aspects within the inflammatory cascade and holds great significance for the development of novel anti-inflammatory drugs. Although conventional anti-inflammatory drugs demonstrate short-term benefits, their long-term use may compromise tendon structural integrity and increase the risk of tendon rupture. In recent years, novel treatment approaches based on biomedical materials have garnered attention in anti-inflammatory therapy for tendon diseases and have shown promising results. For instance, synthetic polymer scaffolds with improved mechanical stability can mimic tendon structure and provide support during the repair process. These scaffolds can be combined with bioactive substances, such as anti-inflammatory drugs, to achieve localized release and suppress inflammation. Additionally, gene therapy represents a potential treatment strategy, involving the delivery of specific genes to modulate inflammatory responses. For example, delivering genes that inhibit the NF-κB signaling pathway can alleviate inflammation.

Overall, studying the inflammatory pathways of tendon diseases is crucial for understanding the mechanisms of inflammation in tendon repair processes. Interventions targeting specific signaling pathways can suppress excessive activation of inflammatory responses and provide new therapeutic strategies for tendon disease treatment. However, further research is needed to validate the effectiveness and safety of these strategies and provide more evidence for clinical applications.

To enhance tendon-to-bone healing and promote bone defect regeneration, various approaches have been attempted, with stem cell-related therapies being the preferred option. Stem cells possess multipotent differentiation capabilities and have been widely applied in regenerative medicine. However, their safety and ethical concerns limit their clinical applications. Recent studies have discovered the biological efficacy of stem cell secretome. EVs derived from human mesenchymal stromal cell secretomes show promise in regulating inflammation and reducing fibrous adhesion. Thus, local application of these EVs may enhance rotator cuff repair outcomes.

Fibrosis processes commonly occur following rotator cuff injuries, leading to structural and functional damage to the tendon. Research emphasizes the crucial role of MSC-derived EVs in treating tendon injuries, particularly their anti-fibrotic and immunomodulatory mechanisms. EVs positively influence tendon healing by regulating inflammatory responses, reducing fibrotic reactions, and promoting cell proliferation and differentiation. MSC-EVs possess anti-fibrotic capabilities, inhibiting the expression of fibrosis-related genes, reducing excessive collagen deposition and scar formation, and promoting normal tendon repair and regeneration. In recent years, extensive research has demonstrated the tremendous potential of MSC-EVs (EVs derived from MSCs) in injury repair and regeneration, displaying effectiveness in clinical applications. MSC-EVs exert their effects through various mechanisms, including modulating macrophage polarization, promoting angiogenesis, enhancing bone formation, and inhibiting tunnel bone resorption. Among these mechanisms, immunomodulation and anti-fibrotic actions are particularly important. MSC-EVs participate in immune responses, regulate macrophages involved in inflammation, promote neovascularization, and inhibit scar formation by stimulating fibroblasts and modulating relevant signaling pathways. These characteristics make MSCs an ideal tool for improving tendon-to-bone healing and scarless repair. However, these findings still require further validation through clinical research.

While the effectiveness of MSC-EVs in rotator cuff tendon–bone healing has been confirmed, their mechanisms of action require further investigation to develop more efficient MSC-EV products for clinical translation. The clinical translation of EV therapy associated with MSC secretion still faces challenges. Firstly, there is currently no gold standard protocol for high-yield isolation of EVs. Secondly, the optimal frequency of EV injections remains uncertain, and it is unclear if multiple injections are more effective than a single injection. Extensive research is required to establish the optimal dosage and injection frequency. Thirdly, EVs can be secreted by various types of cells, each with different functionalities. Further research is needed to compare the differences between EVs from different cell sources in a clinical setting to provide the best choice for tendon–bone healing. Fourthly, most EV treatment trials have focused on small animal models, as the tendon–bone healing process in these models is faster than in human patients. Further research is needed to test the effectiveness and safety of this application in large animals to demonstrate its clinical feasibility. Fifthly, owing to the diversity and complexity of EV components, further exploration is needed to identify specific substances within the exosomes that can improve tendon–bone healing. Finally, further research is needed to optimize the targeting of exosomes as drug delivery vehicles, which may address challenges encountered in cell-based therapies and provide a new “cell-free” comprehensive treatment approach.

In addition, the application of biomaterials and the development of innovative treatment strategies hold great potential for improving the management of tendon injuries. Further research in these areas is necessary to provide more effective treatment options for tendon injury patients. Overall, tendon healing and integration with the bone remain complex processes, and ongoing research aims to deepen our understanding and develop more efficient treatment strategies. The combination of biological and mechanical interventions shows promise in promoting tendon healing and improving clinical outcomes for tendon injury patients. Thus, understanding the inflammatory microenvironment and its role in tendon–bone healing is crucial for optimizing healing outcomes. Balancing inflammatory responses and exploring treatment strategies that can promote beneficial aspects while inhibiting harmful aspects are key considerations. Continuous research on the biological mechanisms of cytokines, the use of biomaterials, and the development of innovative treatment approaches will help improve the management of tendon injuries and enhance tissue repair quality.

Therefore, further in-depth research is needed to address these limitations. In recent years, a growing body of evidence has highlighted the critical role of inflammatory responses in tendon diseases. New ideas have been introduced in the study of tendon disease inflammation, such as the use of single-cell sequencing and spatial transcriptomics to identify key cellular phenotypes driving the pathogenic mechanisms, targeting these pathogenic cytokines and signaling pathways. Thus, a thorough understanding of the mechanisms of inflammation is essential for developing safe and controllable treatment methods to improve tendon healing.

## 9 Conclusions

In summary, gaining an understanding of the role of inflammation in tendon–bone healing is crucial. Tendon injuries are complex, and inflammation plays a key role in the healing process. Striking the delicate balance between beneficial and detrimental factors is essential for optimal outcomes. Investigating the interactions between different cell types and the ECM is vital for tendon healing. Macrophages, inflammatory cytokines, chemokines, growth factors, and fibroblasts play critical roles in the inflammatory microenvironment. Modulating macrophage activity, either inhibiting or enhancing it, can contribute to improved healing outcomes. The NF-κB pathway is an important signaling pathway, and suppressing sustained inflammatory responses is crucial to prevent excessive recruitment of pro-inflammatory mediators. Biomedical materials, gene therapy, and EVs derived from stem cells offer promising therapeutic strategies. However, further research is needed to validate the clinical efficacy and safety of these approaches. A comprehensive understanding of the inflammatory pathways is essential for the development of more effective treatment options for tendon injuries.

## Glossary

RCTs: rotator cuff tears
ACL: anterior cruciate ligament
ECM: extracellular matrix
BTI: bone-to-tendon interface
IL-6: interleukin-6
IL-1β: interleukin-1 beta
bFGF: basic fibroblast growth factor
BMPs: bone morphogenetic proteins
TGF-β: transforming growth factor-beta
VEGF: vascular endothelial growth factor
IL-1: interleukin-1
TNF-α: tumor necrosis factor-alpha
PDGF-CC: platelet-derived growth factor-CC
T2DM: type 2 diabetes mellitus
ROS: reactive oxygen species
NETs: neutrophil extracellular traps
NADPH oxidase: nicotinamide adenine dinucleotide phosphate oxidase
IL-23: interleukin-23
IL-22: interleukin-22
Th2 cells: type 2 T helper cells
Th1 cells: type 1 T helper cells
FGF2: fibroblast growth factor 2
NGF: nerve growth factor
CGRP: calcitonin gene-related peptide
PAR-2: protease-activated receptor 2
NK cells: natural killer cells
TLRs: Toll-like receptors
PAMPs: pathogen-associated molecular patterns
DAMPs: damage-associated molecular patterns
NF-kB: nuclear factor kappa-light-chain-enhancer of activated B cells
VEGF-A: vascular endothelial growth factor-A
IL-8: interleukin-8
IL-18: interleukin-18
CKLF-1: chemokine-like factor 1
COX-1: cyclooxygenase-1
IFN-γ: interferon-gamma
IL1-β: interleukin-1 beta
iNOS: inducible nitric oxide synthase
NLRP3: NOD-like receptor family pyrin domain containing 3
Tregs: regulatory T cells
TLR4: Toll-like receptor 4
NSAIDs: nonsteroidal anti-inflammatory drugs
GDF7: growth differentiation factor 7
TSCs: tendon stem cells
MSC: mesenchymal stem cells
ASC: adipose-derived stem cells
IL-1b: interleukin-1 beta
EVs: extracellular vesicles
Ex: exosomes
MVs: microvesicles
RNA: ribonucleic acid
MSC-EVs: MSC-derived extracellular vesicles
hUC-MSC-sEVs: human umbilical cord-derived MSC-secreted extracellular vesicles
MRI: magnetic resonance imaging
PRP: platelet-rich plasma
HMGB1: high mobility group box 1.
